# Ethinylestradiol and Levonorgestrel as Active Agents in Normal Skin, and Pathological Conditions Induced by UVB Exposure: In Vitro and In Ovo Assessments

**DOI:** 10.3390/ijms19113600

**Published:** 2018-11-14

**Authors:** Dorina Coricovac, Claudia Farcas, Cristian Nica, Iulia Pinzaru, Sebastian Simu, Dana Stoian, Codruta Soica, Maria Proks, Stefana Avram, Dan Navolan, Catalin Dumitru, Ramona Amina Popovici, Cristina Adriana Dehelean

**Affiliations:** 1 Faculty of Pharmacy, “Victor Babeș” University of Medicine and Pharmacy, 300041 Timișoara, Romania; dorinacoricovac@umft.ro (D.C.); farcas.claudia@umft.ro (C.F.); simu.sebastian@umft.ro (S.S.); codrutasoica@umft.ro (C.S.); proks.maria@yahoo.ro (M.P.); stefana.avram@umft.ro (S.A.); cadehelean@umft.ro (C.A.D.); 2 Faculty of Medicine, “Victor Babeș” University of Medicine and Pharmacy, 300041 Timișoara, Romania; nicacristian500@gmail.com (C.N.); stoian.dana@umft.ro (D.S.); navolan@yahoo.com (D.N.); dumcatal@yahoo.com (C.D.); 3 Faculty of Dentistry, “Victor Babeș” University of Medicine and Pharmacy, 300041 Timișoara, Romania; ramona.popovici@umft.ro

**Keywords:** ethinylestradiol, levonorgestrel, keratinocytes, fibroblasts, melanocytes, melanoma, ultraviolet radiation

## Abstract

The link between melanoma development and the use of oral combined contraceptives is not fully elucidated, and the data concerning this issue are scarce and controversial. In the present study, we show that the components of oral contraceptives, ethinylestradiol (EE), levonorgestrel (LNG), and their combination (EE + LNG) ± UVB (ultraviolet B radiation) induced differential effects on healthy (human keratinocytes, fibroblasts, and primary epidermal melanocytes, and murine epidermis cells) and melanoma cells (human—A375 and murine—B164A5), as follows: (i) at low doses (1 µM), the hormones were devoid of significant toxicity on healthy cells, but in melanoma cells, they triggered cell death via apoptosis; (ii) higher doses (10 µM) were associated with cytotoxicity in all cells, the most affected being the melanoma cells; (iii) UVB irradiation proved to be toxic for all types of cells; (iv) UVB irradiation + hormonal stimulation led to a synergistic cytotoxicity in the case of human melanoma cells—A375 and improved viability rates of healthy and B164A5 cells. A weak irritant potential exerted by EE and EE + LNG (10 µM) was assessed by the means of a chick chorioallantoic membrane assay. Further studies are required to elucidate the hormones’ cell type-dependent antimelanoma effect and the role played by melanin in this context.

## 1. Introduction

The admission in use of the oral hormonal contraceptives in the 1960s marked a new period in the pregnancy prevention methods [1]. Despite the progress recorded in the field of contraception methods (intrauterine devices, weekly transdermal patches, long-acting hormone-releasing implants, and monthly vaginal rings), combined oral contraceptives continues to be the most preferred form of reversible hormonal birth control [2]. These pills consist of an estrogen and a progestin component [3]. The estrogen component existent in most of the past and current combined oral contraceptives is 17α-ethinylestradiol (EE), a semisynthetic estrogen, obtained in 1938 by substitution of estradiol at C17 with an ethinyl group, and is described as the most widely used orally bioactive estrogen [2,3,4,5]. Levonorgestrel (LNG—13β-ethyl-17α-ethynyl-17β-hydroxy-4-gonen-3-one) is a second-generation synthetic progestogen, a component of oral contraceptives, and included in “category X” of the drugs forbidden in pregnancy [6].

Even after more than five decades of use by an impressive number of women worldwide (hundreds of millions), the safety issues of oral contraceptives still represents a serious matter of concern. The cardiovascular side-effects related to estrogen component were decreased by the gradual decline of ethinylestradiol dosage from 50 to 20 and even 15 µg, whereas the carcinogenic potential of these pills is still debatable [4,7].

Past and recent studies reported that the role of oral contraceptives in the development of cancer (incompletely elucidated) might be considered tissue-type dependent, since after their use, a protective effect was observed by decreasing the risk of endometrial, ovarian, and colorectal cancers, and an increased risk of breast, cervical, and liver cancer was reported [1,7]. In the last few years, growing clinical and experimental evidence regarding the implication of estrogens in skin cancers, and mainly in melanoma development was gathered, but the data on this subject are still scarce and controversial [8,9,10]. A recent population-based case-control study presented data that support the hypothesis that a long period of use of oral contraceptives (especially with high concentrations of estrogen > 50 mg) is associated with an increased risk of a keratinocyte-derived cancer (squamous cell carcinoma—SCC and basal cell carcinoma—BCC) occurrence [11].

Melanoma is characterized as one of the most immunogenic malignancies, based on its histological, clinical, and genetic heterogeneity that leads to drug resistance to current therapies, reduced tumor regression and survival rates, and converts it into a very demanding challenge [12]. To elucidate the complexity of melanoma growth and progression, novel theories were suggested, consisting of a new approach that describes melanoma as a hormone-related cancer type [9]. This approach might be supported by the following arguments: (i) a gender disparity was observed in melanoma (a reduced incidence, better prognosis and increased survival outcome in female population) [13]; (ii) the estrogen receptors (mainly estrogen receptor β—ERβ) are located in epidermal keratinocytes, dermal fibroblasts, and melanocytes, receptors that mediate key signaling pathways involved in cell proliferation and differentiation, wound healing, skin immune response, and protection against skin photoaging [13,14,15,16]; (iii) estrogens play an important role in cell pigmentation activity [17], an impairment of this function leading to the development of melasma (a disorder of melanogenesis characterized by the presence of an increased number of active melanocytes), or even melanoma [18]; (iv) stimulation of ERβ inhibits the proliferation and migration of malignant cells; the loss of ERβ in melanoma and estrogen-related tumors causes a diminished inhibition of malignant melanocytes proliferation, which in turn is stimulated by ERα [19]; (v) progesterone receptors were found in some keratinocytes and in the nuclei of basal cells [20], and controversial results (stimulatory vs. inhibitory effects) were obtained in terms of melanocyte proliferation as a result of progesterone activity [17]. However, the role played by estrogens and progestins in melanoma development is still uncertain; a large study published in 2017 revealed that estrogens alone increase the risk of melanoma, while the estrogen–progestin combined therapy exerted an opposite activity in terms of melanoma development [21]. Another recently published study (2017) showed that the expression of estrogen and progesterone receptors is a sporadic phenomenon in some cases of malignant melanoma [10]. Natale et al. reported that endogenous estrogen and progesterone mutually regulate melanin synthesis through membrane-bound receptors, even in the absence of classical estrogen or progesterone receptors [22].

One of the main risk factors associated with melanoma development is natural or artificial ultraviolet radiation [23] while sex steroid hormones, both endogenous and exogenous are considered as secondary risk factors [21,23]. The harmful activity of ultraviolet radiation on the human body, recognized as a carcinogen agent, may remain inactive for many years until exposure to certain promoters. Hormones and oncogenes are the most eloquent examples of such agents, with estrogens being labeled both as mutagenic agents of the DNA, and as promoters of cell specific alterations [24,25].

An interplay between female sex steroid hormones and UVB irradiation was discussed within the literature, but a direct link between these agents and the development of melanoma has not yet been found. The regular intake of a levonorgestrel–ethinylestradiol combination led to a phototoxic reaction at the skin level, due to a high absorption of UVB and UVA radiation, both for the two substances and for their combination [26], respectively. In vivo estrogen and UVB exposure produced inflammatory mediators in the skin, and thus led to an improper physiological skin response to UV radiation [27]; thus, UVB radiation may induce differential effects of estrogens on the skin [28]. On the contrary, estrogens showed a protective role at the skin level against UVB chronic irradiation, by employing various mechanisms [29].

Another key player in melanoma development is melanogenesis, a metabolic pathway that is specific for both normal and malignant melanocytes, that interferes with melanoma cell behavior and their surrounding environment [30,31,32]. Melanin is considered to be “a double edge sword” by acting as a protector of melanocytes against UVB deleterious effects and oxidative stress, and on the other hand, a deregulated melanogenesis leads to an increased melanoma resistance to therapy (melanogenesis intermediates exert mutagenic, genotoxic and immunosuppressive properties, and induce hypoxia by upregulating HIF-1α expression) [31,32]. Moreover, it was proven that amelanotic melanomas exhibit a higher susceptibility to radiotherapy as compared to melanotic melanomas, and the overall survival of the patients with amelanotic melanomas is increased [33,34].

Taken together, with all of the information stated above, we could conclude that the current data regarding the association of oral combined hormonal therapy, UV radiation, and skin malignancies is still poor. In this study, we focused on the evaluation of the cytotoxic profile of ethinylestradiol (EE), levonorgestrel (LNG), and their association (EE + LNG), with and without UVB irradiation, on healthy cell lines (human keratinocytes, fibroblasts, and primary epidermal melanocytes, and mouse epidermis cells) and tumor cell lines (human and murine melanoma) by in vitro (viability, migration and proliferation) and in vivo (HET-CAM) techniques.

## 2. Results

### 2.1. Ethinylestradiol and Levonorgestrel ± UVB Irradiation Induced Differential Effects on Healthy Cell and Tumor Cell Viability

**Figure 1:**
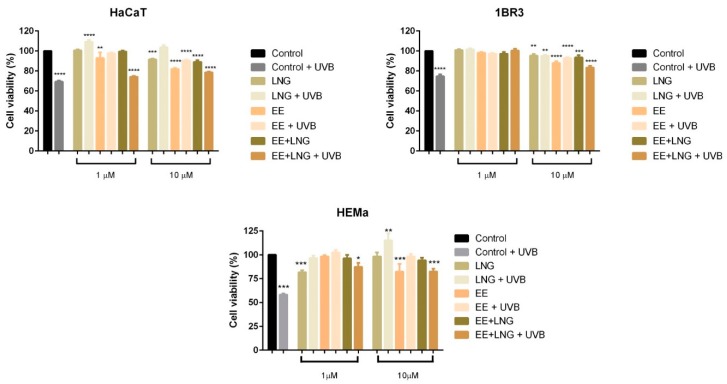
The effect of test compounds (1 and 10 µM) ± UVB irradiation on HaCaT—human keratinocytes, 1BR3—human skin fibroblasts and HEMa—primary human epidermal melanocytes viability at 24 h post-stimulation. The results are expressed as cell viability percentage (%) normalized to control cells. The data represent the mean values ± SD of three independent experiments. One-way analysis of variance (ANOVA) analysis was applied to determine the statistical differences followed by Tukey’s multiple comparisons test (* *p* < 0.05; ** *p* < 0.01; *** *p* < 0.001, **** *p* < 0.0001). EE: ethinylestradiol; LNG: levonorgestrel.

**Figure 2:**
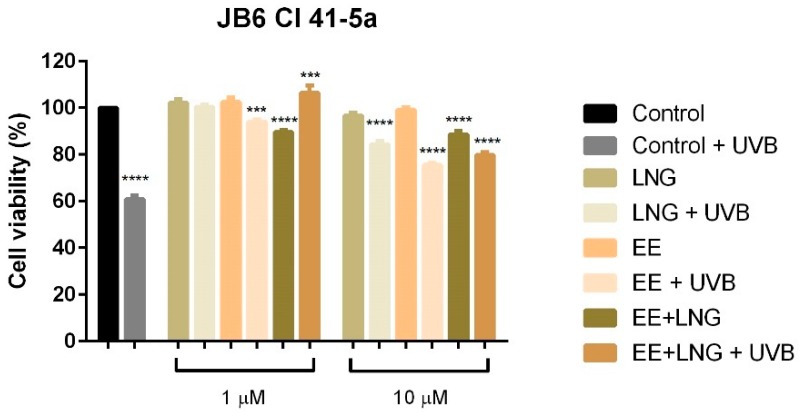
The effect of test compounds (1 and 10 µM) ± UVB irradiation on JB6 Cl 41-5a cell viability at 24 h post-stimulation. The results are expressed as cell viability percentage (%) normalized to control cells. The data represent the mean values ± SD of three independent experiments. One-way ANOVA analysis was applied to determine the statistical differences followed by Tukey’s multiple comparisons test (*** *p* < 0.001, **** *p* < 0.0001).

To assess the effect induced by test compounds (EE, LNG and EE + LNG) on healthy human and murine skin (HaCaT, 1BR3, HEMa and JB6 Cl 41-5a) cells, and melanoma (A375 and B164A5) cell viability in the presence/absence of UVB irradiation, we performed the Alamar blue assay. Irradiation of HaCaT, 1BR3, HEMa and JB6 Cl 41-5a cells with UVB (40 mJ/cm^2^) resulted in a significant reduction of cells viability (66.30% viable HaCaT, 74.75% viable 1BR3, 58.25% viable HEMa, and 60.85% viable JB6 Cl 41-5a, respectively) as compared to control cells (unirradiated cells) (Figure 1 and Figure 2). Stimulation of healthy cells with EE (1 and 10 µM) for 24 h led to the following results: (i) HaCaT cells—a slight decrease of viability in a dose-dependent manner (92.90% at 1 µM and 82.01% at 10 µM), (ii) 1BR3 cells—88.04% viable cells at 10 µM, (iii) HEMa cells—82.25% viable cells at 10 µM, and (iv) JB6 Cl 41-5a cells—the viability was not affected as compared to control cells (unstimulated cells) (Figure 1 and Figure 2). Levonorgestrel had no influence on HaCaT, 1BR3, and JB6 Cl 41-5a cell viability after 24 h stimulation at the lowest concentration tested—1 µM, whereas at 10 µM it was recorded a decrease <10% in the case of HaCaT cells and <5% in the case of 1BR3 and JB6 Cl 41-5a. In the case of HEMa cells, the effect of levonorgestrel was somehow reversed as compared to the other healthy cells: the lowest concentration—1 µM decreased cells viability (81.69%), whereas at 10 µM, no toxicity was observed. A combination of EE + LNG induced a decline of JB6 Cl 41-5a viability (around 10%) at both tested concentrations (1 and 10 µM), with HaCaT, 1BR3, and HEMa cells being affected only at the highest concentrations (<10% decrease for HaCaT and 1BR3 and <5% decrease for HEMa) (Figure 1 and Figure 2).

The lowest viability rates were observed in the groups of cells that were irradiated with UVB and stimulated with the combination of hormones—EE + LNG (at 10 µM); still, these viability percentages were higher as compared to the ones recorded for the cells that were only UVB-exposed (HaCaT: 78.55% vs. 69.30%; 1BR3: 83.31% vs. 74.75%, HEMa: 82.46% vs. 58.25%, and JB6 Cl 41-5a: 79.83% vs. 60.85%), what might indicate a recovery effect induced by EE + LNG stimulation after UVB noxious effects on healthy skin cells (see Figure 1 and Figure 2).

Similar experimental conditions to the ones described for healthy cells were applied for A375 and B164A5 melanoma cells in order to evaluate the effects induced by test compounds (1 and 10 µM) ± UVB irradiation on cells viability in a 24 h frame.

**Figure 3:**
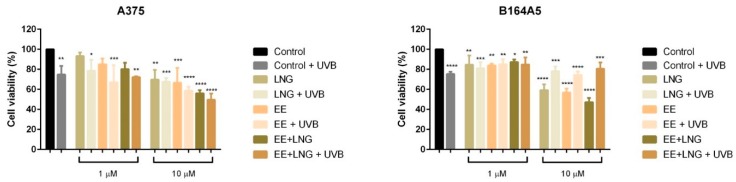
The effect of test compounds (1 and 10 µM) ± UVB irradiation on A375—human melanoma and B164A5—murine melanoma cell viability at 24 h post-stimulation. The results are expressed as a cell viability percentage (%) normalized to control cells. The data represent the mean values ± SD of three independent experiments. One-way ANOVA analysis was applied to determine the statistical differences followed by Tukey’s multiple comparisons test (* *p* < 0.05; ** *p* < 0.01; *** *p* < 0.001, **** *p* < 0.0001).

Results showed that UVB irradiation of human and murine melanoma cells determined a significant decrease of cell viability (around 75%) as compared to control cells (unirradiated cells) (Figure 3). Both EE and LNG induced a dose-dependent decline of A375 and B164A5 cell viability, but the lowest viability percentage was calculated for the EE + LNG at the highest concentration used—10 µM (56% for A375 and 47.23% for B164A5). Exposure to UVB radiation followed by stimulation with EE, LNG, or EE + LNG led to a significant dose-dependent decrease of A375 cell viability percentage, decrease that was considerably stronger as compared to the effects induced by each test compound/UVB alone, what might lead to the conclusion that the used agents had a synergistic cytotoxic effect on A375 cells (EE vs. EE + UVB: 66.54% vs. 58.72%; LNG vs. LNG + UVB: 69.78% vs. 67.59%; EE + LNG vs. EE + LNG + UVB: 56% vs. 49.69%). In the case of B164A5 cells, UVB irradiation followed by stimulation with test compounds produced an inverse effect as compared to A375; namely, an increase of the cells’ viability as compared with the values obtained for each test compound (EE vs. EE + UVB: 56.84% vs. 74.46%; LNG vs. LNG + UVB: 59.27% vs. 78.06%; EE + LNG vs. EE + LNG + UVB: 47.23% vs. 80.59%) (Figure 3). A similar effect as the one described for B164A5 was observed in the case of pigmented human melanoma cells—RPMI-7951 (see Figure S1).

### 2.2. Ethinylestradiol and Levonorgestrel ± UVB Irradiation Triggered Apoptosis in A375 and B164A5 Melanoma Cells

Based on the results described above, according to which the test compounds (EE, LNG, EE + LNG) ± UVB significantly decreased the viability of human and murine melanoma cells, it was verified if the cells death was achieved via apoptosis; the analysis was performed using an annexin V/PI (propidium iodide) apoptosis detection kit. The cells were stimulated for 24 h with EE, LNG and EE + LNG (1 and 10 µM) ± UVB irradiation.

**Figure 4:**
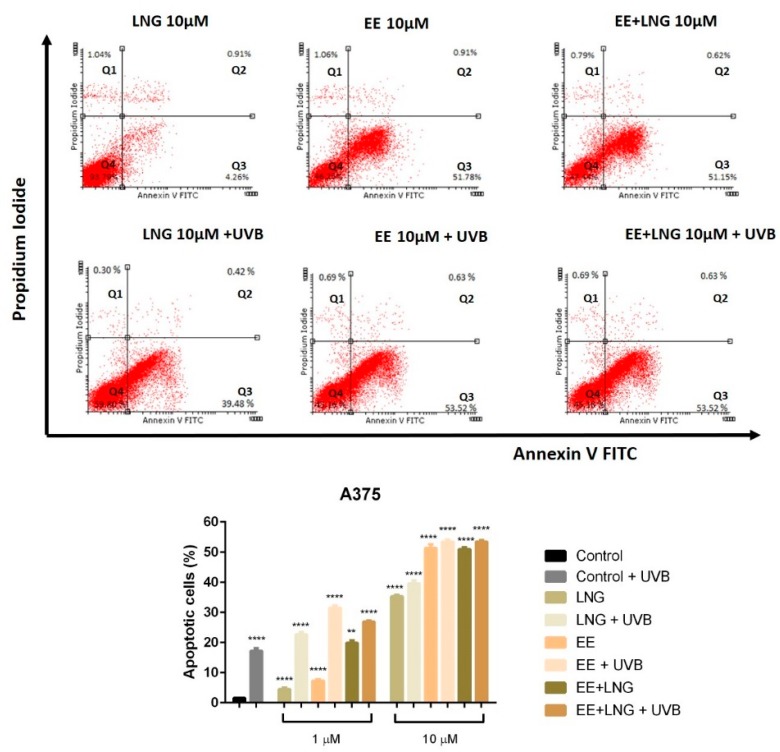
Representative dot plots of the apoptotic events induced by test compounds (EE, LNG, EE + LNG—10 µM) ± UVB irradiation in A375 human melanoma cells after a 24 h stimulation. The cells status was analyzed by a FACS technique where: Q4—viable cells, Q3—early apoptotic cells, Q2—late apoptotic cells and Q1—necrotic cells. The graph represents the percentage of early apoptotic A375 cells. The results are expressed as apoptotic cell percentage (%) normalized to control cells. The data represent the mean values ± SD of three independent experiments. One-way ANOVA analysis was applied to determine the statistical differences, followed by Tukey’s multiple comparisons test (** *p* < 0.01; **** *p* < 0.0001).

A dose-dependent apoptotic activity was noticed in the case of both cell lines. As compared to control cells (unstimulated cells), the strongest apoptotic effect on non UVB-irradiated A375 human melanoma cells was induced by EE and EE + LNG at the highest concentrations tested—10 µM); the percentage of early apoptotic cells was 51.78% for EE and 51.15% for EE + LNG (Figure 4), data that confirm the results obtained for viability assessment. At the same concentration, LNG alone exerted a lower pro-apoptotic activity. UVB exposure of A375 cells, followed by addition of 1 µM of test compounds led to a significantly increased percentage of early apoptotic cells as follows: 22.62% for LNG; 31% for EE and 27% for EE + LNG. UVB irradiation combined with the highest concentration—10 µM of test compounds triggered percentages of the early apoptotic population similar to the ones recorded for the test compounds in non UVB-exposed cell population (Figure 4).

**Figure 5:**
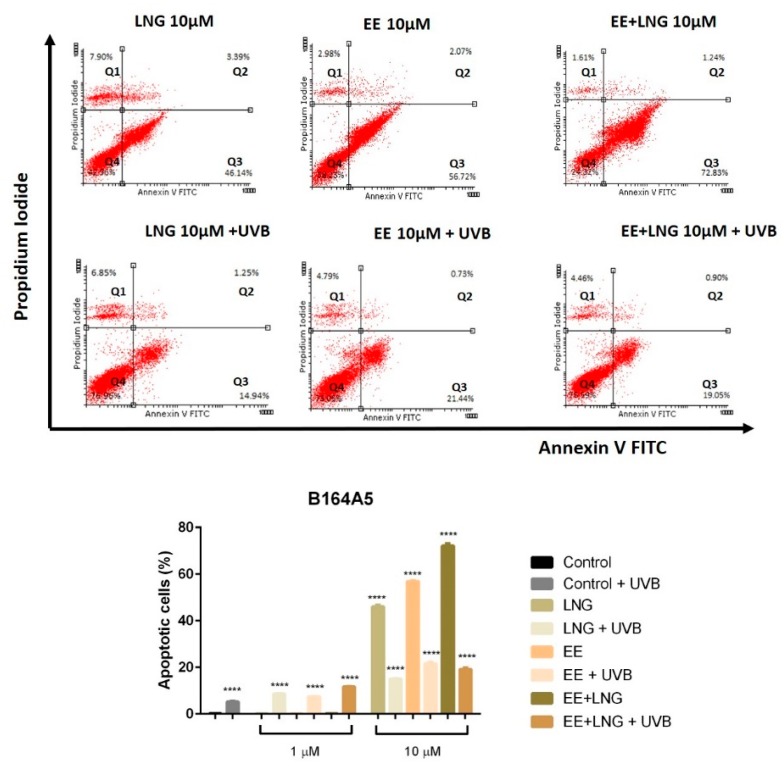
Representative dot plots of the apoptotic events induced by test compounds (EE, LNG, EE + LNG—10 µM) ± UVB irradiation in B164A5 murine melanoma cells after a 24 h stimulation. The cells status was analyzed by a FACS technique where: Q4—viable cells, Q3—early apoptotic cells, Q2—late apoptotic cells and Q1—necrotic cells. The graph represents the percentage of early apoptotic B164A5 cells. The results are expressed as apoptotic cell percentage (%) normalized to control cells. The data represent the mean values ± SD of three independent experiments. A one-way ANOVA analysis was applied to determine the statistical differences followed by Tukey’s multiple comparisons test (**** *p* < 0.0001).

In Figure 5 was depicted the effect of the test compounds ± UVB irradiation on B164A5 murine melanoma cells apoptotic process; the highest concentration tested—10 µM induced a drastic decrease of cell viability and caused the most significant pro-apoptotic effect with a maximum of 72.83% for EE + LNG. After UVB exposure and 1 µM of test compounds, one can notice the absence of the pro-apoptotic process and the subsequently increased cell viability. At 10 µM, B164A5 UVB-irradiated murine melanoma cells showed a slight apoptosis induction, with the highest pro-apoptotic level noted for EE (21.44%) (Figure 5).

### 2.3. Ethinylestradiol (EE) and Levonorgestrel (LNG) ± UVB Irradiation Determined Changes in Cells Morphology

**Figure 6:**
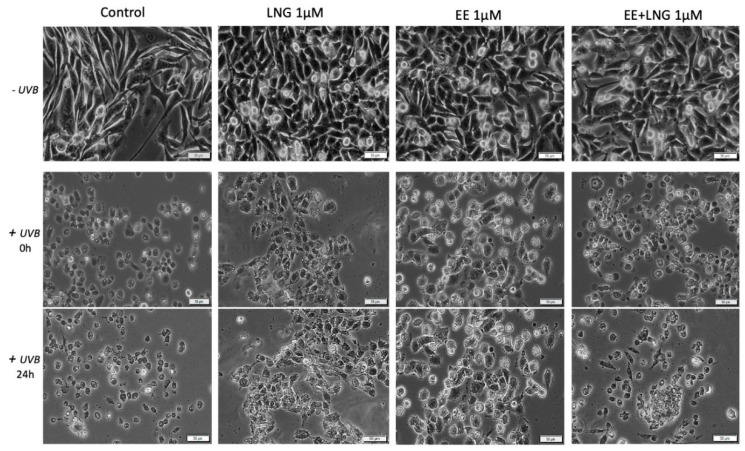
In vitro morphological aspects of human keratinocytes—HaCaT cells, stimulated with levonorgestrel (LNG), ethinylestradiol (EE), and an ethinylestradiol/levonorgestrel combination (EE + LNG), respectively, at a concentration of 1 µM ± UVB irradiation. Scale bars represent 50 µM.

Immortalized human keratinocytes—HaCaT showed no significant morphological changes after stimulation with EE, LNG, and EE + LNG (1 µM). Their shape remained well defined, elongated, and the cells were attached to the culture plate. In contrast, after UVB irradiation, HaCaT cells drastically changed their morphological aspect, becoming round and some of them floating; the most affected cells seemed to be the ones stimulated with EE + LNG. Cell shrinkage was also noticed, and could be considered a sign of early apoptosis, results that are consistent with the data described in the apoptosis assessment section. At 24 h post-exposure to UVB, HaCaT cells stimulated with EE and LNG looked like they began to partially recover, results that confirm the cell viability data (Figure 6).

**Figure 7:**
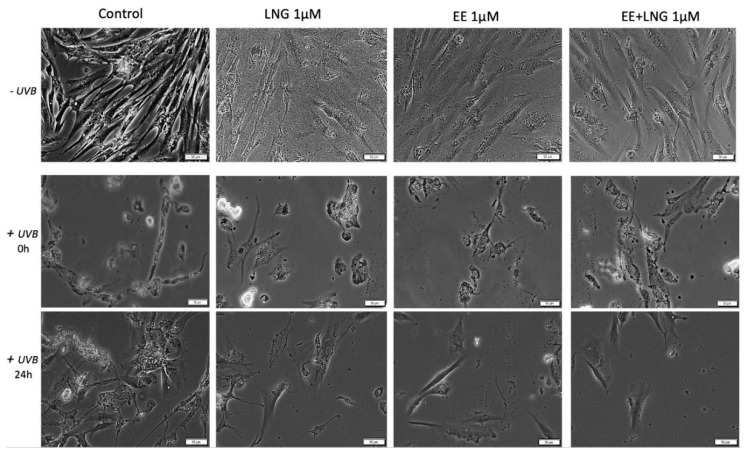
In vitro morphological aspect of human fibroblasts—1BR3 cells, stimulated with levonorgestrel (LNG), ethinylestradiol (EE), and an ethinylestradiol/levonorgestrel combination (EE + LNG), respectively, at a concentration of 1 µM ± UVB irradiation. Scale bars represent 50 µM.

In the case of human skin fibroblasts—1BR3, the results were similar as for HaCaT cells, with no changes in cells shape following stimulation with EE, LNG, and EE + LNG were noticed; the cells morphology preserving the same needle-like shape and the same confluence as the control cells. Control cells exposed to UVB showed various degrees of cell shrinkage; but after stimulation with EE, LNG, and EE + LNG, respectively, cells began to regain their initial morphological aspect with bright and compact cell margins; however, the colonial morphology was not entirely recovered after 24 h but recovery signs in cells shape were detected (Figure 7).

**Figure 8:**
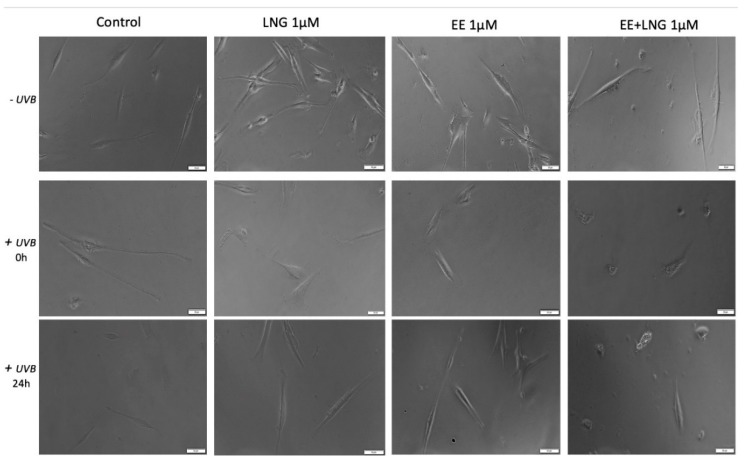
In vitro morphological aspects of primary human epidermal melanocytes—HEMa cells, stimulated with levonorgestrel (LNG), ethinylestradiol (EE), and ethinylestradiol/levonorgestrel combination (EE + LNG), respectively, at a concentration of 1 µM ± UVB irradiation. Scale bars represent 50 µM.

Stimulation of primary human epidermal melanocytes—HEMa with EE and LNG (1 µM) had no effects on cells morphology, the cells were adherent to the culture plate and presented a needle-like/dendritic-like shape similar to control cells. EE + LNG induced a slight modification of HEMa cells morphology. UVB irradiation influenced the melanocytes’ shape and their confluence, and the association with EE + LNG seemed to be the most noxious. At 24 h post-exposure to UVB, HEMa cells stimulated with EE and LNG partially gained their initial form, results that confirm the cell viability data (Figure 8).

**Figure 9:**
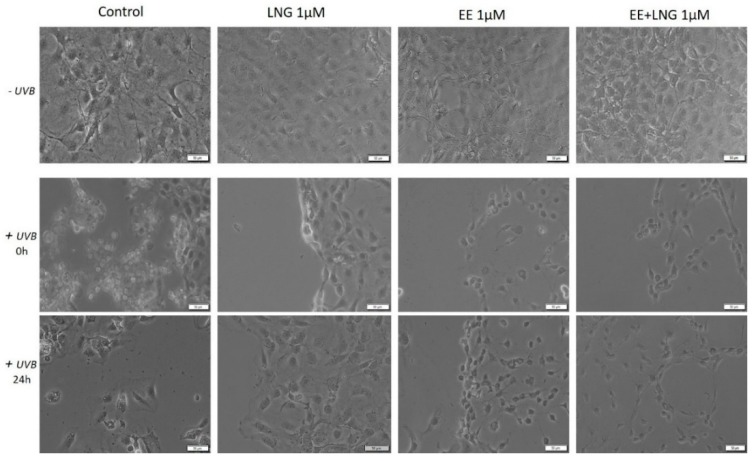
In vitro morphological aspect of mice epidermis—JB6 Cl 41-5a cells, stimulated with levonorgestrel (LNG), ethinylestradiol (EE), and an ethinylestradiol/levonorgestrel combination (EE + LNG), respectively, at a concentration of 1 µM ± UVB irradiation. Scale bars represent 50 µM.

Murine epidermis JB6 Cl 41-5a cells showed a good confluence in the absence of UVB radiation and the test compounds did not perturb the shape of the cells; whereas after UVB exposure, the cells stimulated with test compounds seemed to be protected by UVB deleterious effects, and only minor changes were observed in the group stimulated with EE. The control cells exposed to UVB were most affected, displaying a low level of confluence and major changes of their morphological aspects, characteristics that were partially recovered after 24 h post-irradiation (Figure 9).

**Figure 10:**
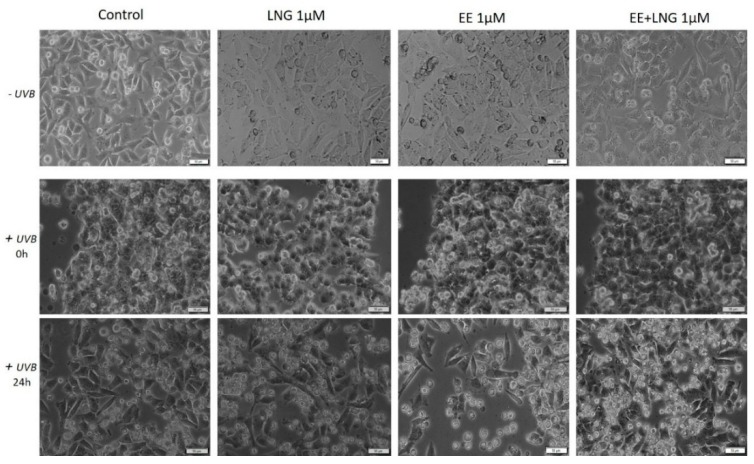
In vitro morphological aspect of human melanoma—A375 cells, stimulated with levonorgestrel (LNG), ethinylestradiol (EE), and an ethinylestradiol/levonorgestrel combination (EE + LNG), respectively, at a concentration of 1 µM ± UVB irradiation. Scale bars represent 50 µM.

Taking into consideration the pro-apoptotic effect of the test compounds on human and murine melanoma cells, the impact of these compounds on melanoma cells morphology was monitored by light microscopy. In the case of A375, the control cells (unstimulated and unirradiated cells) displayed a normal epithelial morphology, with spindle and cobblestone shapes, strongly bounded, adherent to the culture plate, and highly confluent after 24 h. A decrease of A375 control cells confluence was recorded after UVB radiation and some detached and floating cells were noticed. The EE and LNG stimulation of cells exposed to UVB led to some changes in cells’ shape (Figure 10), mainly after EE + LNG treatment; the cells became round and began to detach, indicating the process of apoptosis, the results being in agreement with the reported cell viability data. In the case of pigmented human melanoma cells—RPMI-7951, the test compounds had no impact on cells morphology, but after UVB irradiation, significant changes were observed in all groups (round floating cells), effects that were almost completely reversed after 24 h and test compound stimulation (see Figure S2).

In the case of murine melanoma cells—B164A5, the cells exposed to UVB irradiation seemed to be the most greatly affected in terms of cell morphology, showing a round shape with dendrites and shrinkage. Changes in B164A5 melanoma cells shape were also observed after stimulation with test compounds, in non-UVB irradiated cells.

**Figure 11:**
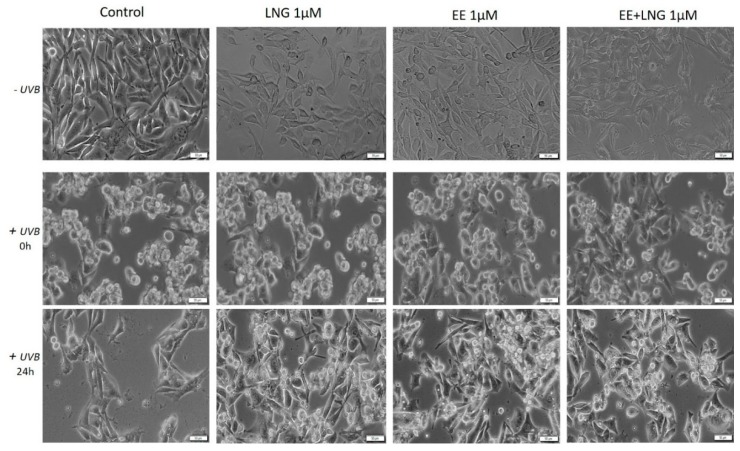
In vitro morphological aspect of murine melanoma—B164A5 cells, stimulated with levonorgestrel (LNG), ethinylestradiol (EE), and an ethinylestradiol/levonorgestrel combination (EE + LNG), respectively, at a concentration of 1 µM ± UVB irradiation. Scale bars represent 50 µM.

On the other hand, B164A5 cells exposed to UVB followed by stimulation with test compounds revealed a confluence increment and minor changes in cells morphology, as shown in Figure 11.

### 2.4. The impact of Ethinylestradiol (EE) and Levonorgestrel (LNG) on Healthy and Tumor Cells Migration and Proliferation

**Figure 12:**
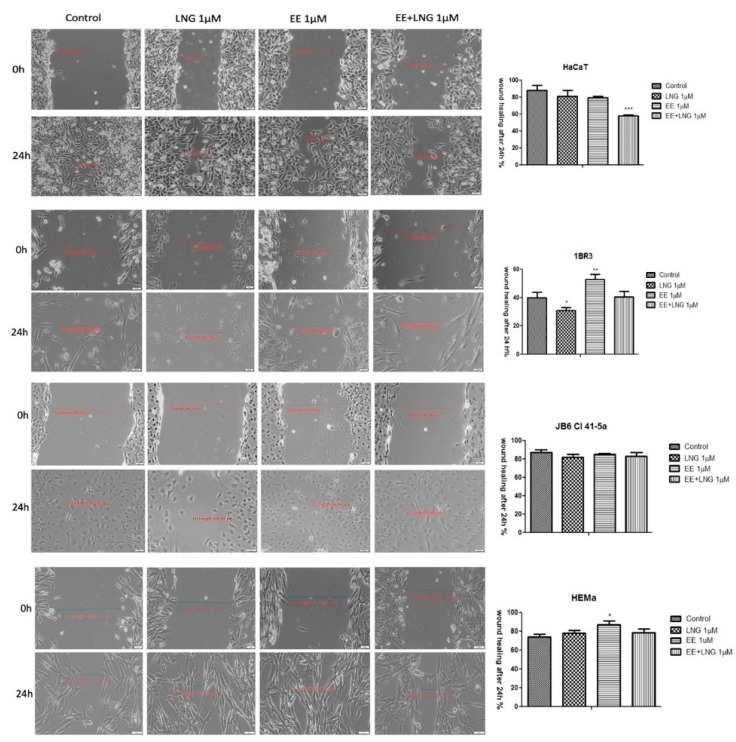
The impact of test compounds (LNG, EE, and EE + LNG—1 µM) on the migratory capacity of healthy skin cell lines (HaCaT—human immortalized keratinocytes, 1BR3—human fibroblasts, JB6Cl415a—mice epidermis, HEMa—primary human epidermal keratinocytes). Wound closure was recorded by bright field microscopy initially—0 h and after 24 h, respectively. Scale bars represent 50 µm. The bar graphs are expressed as percentage of wound closure after 24 h compared to the initial surface. The data represent the mean values ± SD of three independent experiments. One-way ANOVA analysis was applied to determine the statistical differences followed by Tukey post-test (* *p* < 0.05; ** *p* < 0.01; *** *p* < 0.001 vs. control—no stimulation).

Figure 12 displays the migratory activity of the healthy cell lines in the presence of EE, LNG, and EE + LNG. Since at the highest concentration used—10 µM, a cytotoxic and pro-apoptotic effect was observed, and the concentration selected for this assay was 1 µM. LNG stimulation did not interfere with the migration of human and murine healthy skin cells, the wound widths at 24 h being similar to the ones measured for control cells (Figure 12). After EE stimulation, a stimulatory trend in all cell lines could be mentioned as compared to control cells; however, the most significant stimulation was seen with 1BR3 cells (52.37% vs. 40.09% on 1BR3 cells), results that are consistent with cell viability data. The combination of the two hormones—EE + LNG induced an inhibitory effect on HaCaT cells migration, showing a wound closure rate of 58.18%, whereas in the case of 1BR3 and JB6 Cl 41-5a, the effect was a stimulatory one (Figure 12). The very low wound healing rate (40.08%) of 1BR3 control cells was due to their low proliferation ability in specific culture conditions per day. A stimulatory effect on HEMa cells migration was observed after EE and LNG stimulation (the gap was almost covered—mainly after EE) as compared with control cells. Moreover, the combination EE + LNG also augmented the migratory capacity of HEMa cells (Figure 12).

**Figure 13:**
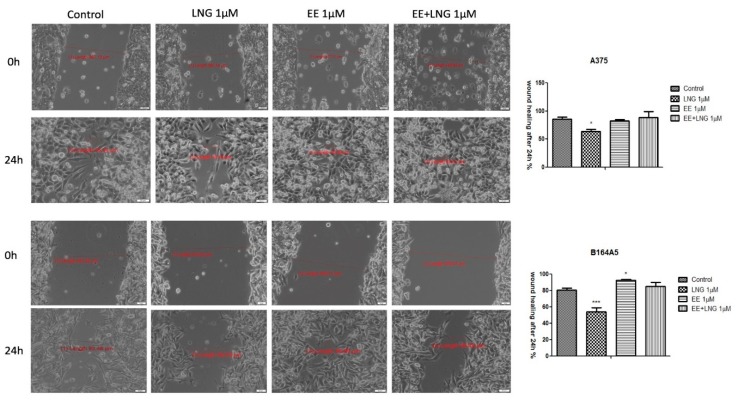
The impact of test compounds (LNG, EE and EE + LNG—1 µM) on migratory capacity of melanoma cell lines (A375—human melanoma cells and B164A5—murine melanoma cells). Wound closure was recorded by bright field microscopy initially and after 24 h, respectively. Scale bars represent 50 µm. The bar graphs are expressed as percentage of wound closure after 24 h compared to the initial surface. The data represent the mean values ± SD of three independent experiments. One-way ANOVA analysis was applied to determine the statistical differences followed by Tukey post-test (* *p* < 0.05; *** *p* < 0.001 vs. control—no stimulation).

The in vitro wound healing assay revealed that the melanoma cells’ (A375—human melanoma, B164A5—murine melanoma) migratory capacity was not inhibited by EE and EE + LNG stimulation (1 µM), moreover, a stimulatory effect could be mentioned; still, the fact that the wound was also covered with some detached cells must be taken into account (Figure 13). For EE, the wound closure rate was 82.81% on human melanoma cells and 85.29% on the murine melanoma cell line. In contrast, the same concentration of LNG (1 M) showed a wound healing rate of only 63.98% in the case of human melanoma cells, and 53.94% in the case of the murine melanoma cell line. Similar results were obtained for human pigmented melanoma cells—RPMI-7951 (see Figure S3).

### 2.5. Irritant Potential Assessment of Ethinylestradiol and Levonorgestrel by the Means of a HET-CAM Assay

The potential toxicity of the test compounds (EE, LNG and EE + LNG) was also assessed in vivo, using the in ovo chick chorioallantoic membrane as a biological environment. The protocol allows the evaluation of the irritant potential of the hormone solutions after topical application. The reaction induced by the tested compounds (Table 1) can be classified according to Luepke, as follows: non-irritant (0–0.9), weak irritant (1–4.9), moderate irritant (5–8.9/9.9), and strong irritant (8.9/9.9–21) [35].

The effects induced by the test compounds, along with the positive (SDS—sodium dodecyl sulfate) and negative (PBS—phosphate saline buffer) controls were registered as photographs representing the upper surface of the chorioallantoic membranes before and after 5 min of contact with the solutions. Prior to the determination of the irritation score, the results recorded for irritation severity were considered. SDS induced major vascular damage on the chorioallantoic membrane. All three endpoints: hemorrhage, coagulation, and lysis, were reported only for SDS.

**Figure 14:**
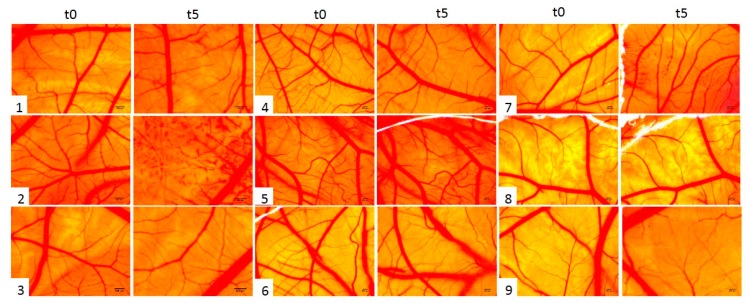
Irritant potential assessment of test compounds using HET-CAM assay: a) stereomicroscope images of the CAMs inoculated with control and test compounds (1—PBS, 2—SDS, 3—DMSO, 4—EE 1 µM, 5—LNG 1 µM, 6—EE + LNG 1 µM, 7—EE 10 µM, 8—LNG 10 µM, 9—EE + LNG 10 µM)—before the application (t0) and after 5 min of contact with the compounds (t5). Scale bars represent 500 µm.

None of the three endpoints were registered for PBS, DMSO 1%, LNG (1 and 10 µM), and the lowest concentration of EE (1 µM). EE (10 µM) showed late and limited signs of hemorrhage or coagulation, and early, though limited signs of vasodilatation. EE + LNG (1 and 10 µM) application induced slight and limited coagulation, in a dose-dependent manner. The highest mean irritation score was recorded for the positive control, SDS, IS = 14.05. Negative and solvent controls were non-irritant. Among the tested hormones, LNG indicated no sign of irritancy even at the highest concentration tested—10 µM. EE induced a weak irritant effect at the highest concentration (Table 1, Figure 14).

SDS induced major vascular damage on the chorioallantoic membrane; after the application of 500 µL solution, a large area was affected by early micro-hemorrhages, coagulation, and later vessel lysis. The death of the specimen was registered within 60 min. For the samples that were non-irritant on the CAM, we registered a viability of more than 24 h. For the samples that induced a weak irritant effect the death was registered within the first 24 h.

All the tested samples induced no damage or merely slight damages on the CAM vascular plexus. LNG was assessed as non-irritant in both concentrations, EE as non-irritant at 1 µM and a weak irritant at 10 µM. Very similar to EE, the combination EE + LNG was considered non-irritant at 1 µM and a weak irritant (however weaker than EE alone) at 10 µM. 

## 3. Discussion

Oral contraceptives have been suspected for a long time to co-participate in some pathways of developing malignant melanoma, but there was no statistical evidence for either exogenous or endogenous hormones clearly increasing the risk of melanoma [36,37,38]. The current scientific data are debatable due to studies that confirm the association between sex hormones and melanoma [11,39,40], while others state the opposite [41,42]. According to Nurses’ Health Study, the risk to developing melanoma is two times higher among women that have used oral contraception for 10 years or more [43]. It was also reported that the use of progesterone alone actuated the growth of melanoma micro-metastases [44]. Another study revealed that only low doses of progesterone (up to 1 µM), similar to the ones used in therapy, are able to stimulate melanoma cell proliferation, while higher doses not only lack such effect but even induce cell cycle arrest and apoptosis [40].

The myriad of biological and environmental factors that are suspected to interfere in melanoma development leaves a wide-open window for hypotheses, and recent studies investigate estrogen-mediated signaling in melanoma (an impairment of estrogen signaling triggers cancer initiation, promotion and progression) [9], by assessing the role of ERα gene promoter methylation or the expression of G protein-coupled ER [10]. Some reports endorse the existence of a direct relationship between skin diseases, endocrinology, and psychological stress [45]. Moreover, a strong interdependence was reported between the stratum corneum integrity, hormonal levels, and UV susceptibility in terms of minimal erythemal doses, therefore suggesting a significant relevance for all these factors in skin pathophysiology [46].

Most of the experimental studies conducted to verify the role of estrogens and progestins in skin biology/pathology employed as test agents: 17β-estradiol (E2) [47,48] and progesterone (endogenous hormones) [40,49], and data regarding the effects of synthetic hormones present in the composition of oral contraceptives, are rather scanty.

All these converging elements determined the implementation of the present study, which was designed to characterize the in vitro and in ovo toxicological profile of the most frequently used synthetic hormones (ethinylestradiol and levonorgestrel) in oral combined contraceptives by applying two different settings: (1) stimulation with EE, LNG, and EE + LNG of healthy skin cells (human keratinocytes, fibroblasts, primary melanocytes, and murine epidermis cells), melanoma cells (human and murine) and chorioallantoic chick membrane; and (2) healthy and tumor cell UVB irradiation (a well-known initiator and promoter of skin cancer), followed by hormone stimulation for 24 h.

The healthy cell lines used in the experiment (HaCaT—immortalized human keratinocytes, 1BR3—human dermal fibroblasts and JB6 Cl 41-5a—mice epidermis cells) were selected based on the following considerations: (i) the presence of estrogen receptors (ERβ) in epidermal keratinocytes and dermal fibroblasts, the main cellular processes at this level being mediated by estrogens; (ii) estrogens exert a stimulatory effect on melanocytes (estrogen-responsive cells) [10]; (iii) keratinocytes and fibroblasts interact in a synergistically manner to maintain a functional epidermis by promoting repair and regeneration post-acute UVB irradiation [50]; (iv) keratinocytes promote UV-induced melanogenesis (tanning) by releasing several pro-pigmenting paracrine growth factors (αMSH, ET-1, and SCF); (v) dermal fibroblasts are involved in the regulation of constitutive pigmentation and in the development of pigmentary disorders [51].

A primary human epidermal melanocytes cell line—HEMa, was also included in the study, taking in consideration the fact that melanogenesis and melanoma development are strongly interrelated [30,31,32,33,34]. In addition, there is evidence that estrogen and progesterone regulate melanin synthesis [22].

Stimulation of healthy cells with EE, LNG, and EE + LNG led to cell type-dependent results, as follows: HaCaT cells were sensitive to EE in a dose-dependent manner, while LNG and EE + LNG affected cells viability only at the highest concentration (10 µM) (see Figure 1); in the case of 1BR3 cells, the tested hormones reduced cells viability only at the highest concentration; HEMa cells were sensitive to LNG (1 µM) and EE (10 µM), whereas EE + LNG did not decrease melanocyte viability (see Figure 1), and the JB6 Cl 41-5a cells proved to be sensitive only after LNG and EE + LNG stimulation (10 µM) (see Figure 2). Altogether, our results indicate that the lowest concentration (1 µM) of the tested hormones and their combination could be considered without significant toxicity on healthy cells viability, but an increased concentration could affect this status (approximately 75–90% viable cells at 10 µM). A decreased percentage of viable HaCaT cells was also reported after stimulation with high concentrations of EE [52], data that are consistent with our results. The endogenous estrogenic hormone, 17β-estradiol stimulated the proliferation of human normal keratinocytes by augmenting the proportion of cells in S phase of the cell-cycle [53]. The different cellular response observed after EE and 17β-estradiol stimulation could be explained by the fact that EE predominantly acts on ERα whereas 17β-estradiol is equally active on both ERα and ERβ [5].

Several studies reported beneficial and protective roles of estrogens in skin biology (augmented wound healing, protection against photoaging, increased epidermal thickness, ameliorated inflammatory pathologies) initiated via ERα (particularly detected in sebocytes) and ERβ (highly expressed in various skin cell types) [13,47]. It was also stated that estrogens intervene in cell migration and the protection of cell integrity by controlling cell morphology and inducing the cytoskeleton reorganization of different normal and tumor cell types: human dermal fibroblasts (actin cytoskeleton reorganization, restoration of cell shape cultivated in desteroidated medium, and protection on cells adhesive strength), glial cells, neurons, endothelial cells, osteoblasts, and carcinoma cells [47]. A stimulatory effect on healthy cells (HaCaT, 1BR3 and JB6 Cl 41-5a) migration was observed after stimulation with EE (1 µM); however, EE + LNG induced a slight inhibition of HaCaT cells migratory capacity, and LNG did not influence this process (Figure 12). No morphological changes of healthy cells were noticed after hormones stimulation (Figure 6, Figure 7, Figure 8 and Figure 9).

Concerning the behavior of sex hormones on healthy cells, a recent study demonstrated that a continuous exposure of melanocytes to estrogen led to an increase in melanin production, while progesterone had inverse effects. Moreover, estrogen-treated melanocytes produced a high amount of melanin for 50 days after hormone removal, but in the case of progesterone the cells returned to their baseline level of melanin immediately. In addition, in the melanocytes treated with estrogen, stimulation with progesterone reversed estrogen effects [48]. Similar results were obtained by Wiedeman et al. [52] data that are in agreement with our results. Poletini and co-workers proved in an elegant study that normal and malignant melanocytes respond different to estradiol stimulation [54].

The second setting proposed in this study that involves UVB irradiation determined significant changes in terms of healthy cell viability and morphology. UVB irradiation (40 mJ/cm^2^) reduced significantly the percentage of HaCaT, 1BR3, HEMa, and JB6 Cl 41-5a viable cells, the highest toxicity being recorded for HEMa—58.25% and JB6 Cl 41-5a cells—60% viable cells (Figure 1 and Figure 2). The low percentage of viable melanocytes could be related to the fact that melanocytes are target cells for UV toxicity by acting as shields for the nuclei and for the other skin cells [54]. During UV irradiation, melanin suffers a photosensitization process that results in the production of reactive oxygen species and the lethal insult of individual cells [30]. The susceptibility of murine epidermis skin (JB6 Cl 41-5a) cells to UVB irradiation could be ascribed to the fact that these cells are isolated from primary cultures of neonatal BALB/c epidermal cells, the newborn mice being the most suitable animal model to develop UV-induced melanoma [12]. Similar results regarding the noxious effect of UVB radiation on keratinocytes and fibroblasts viability were described in other studies, the intensity of the cytotoxic effect being dependent on the UVB dose and the experimental conditions applied [55,56,57,58,59]. A recent study showed that a higher dose of UVB (70 mJ/cm^2^) used was nontoxic for fibroblasts [60]. It is well-known that UV radiation affects human skin at physiological, biological, and molecular levels by generating reactive oxygen species that are responsible for DNA damage, cell cycle arrest, and apoptosis, together with increased matrix metalloproteinase and elastase expression, having as a consequence, wrinkle formation and impaired cell migration [57].

UVB irradiation of healthy cells (HaCaT, 1BR3, HEMa, and JB6 Cl 41-5a), followed by hormone stimulation, led to some interesting results concerning their viability status: an increased viability percentage was recorded in all cell lines after UVB irradiation + EE or LNG (at both 1 and 10 µM) as compared to control UVB-irradiated cells (like the hormones “helped” the cells to recover after UVB damage), whereas UVB irradiation + EE + LNG (at 10 µM) proved to be toxic for all cells, and still less toxic as compared to UVB-irradiated cells (see Figure 1 and Figure 2). The morphological features of the healthy cells changed significantly after UVB irradiation (Figure 6, Figure 7, Figure 8 and Figure 9), with data that are confirmed by other studies in the literature [50,57,58]. The cells also stimulated with test hormones showed a lesser extent of damage; most of them presented characteristics similar with the control unexposed cells, results consistent with the viability data.

Considering the increased interest assigned to a possible link between sex hormones/oral contraceptive use and the development of melanoma, and the gaps existent in this regard, we assessed the impact of EE, LNG, and EE + LNG ± UVB irradiation on human (A375) and murine (B164A5) melanoma cells to provide reliable data concerning the current controversial reports. The test hormones exerted a dose-dependent cytotoxic effect on both A375 and B164A5 melanoma cells, the lowest percentage of viable cells being recorded after stimulation with EE + LNG—10 µM (56% and 47.23%, respectively) (see Figure 3). A considerable number of cells were floating, and this observation determined us to verify the type of cell death induced by the test compounds. An annexin V/PI test confirmed that the test hormones induced apoptosis of melanoma cells, the proapoptotic effect was also dose-dependent, and the strongest activity was triggered by EE + LNG (see Figure 4 and Figure 5). The choice of the two different melanoma cell lines—A375 (human amelanotic cells) and B164A5 (murine melanotic cells) was based on the different response that was recorded in terms of melanoma aggressiveness, overall survival, and anti-melanoma therapies [30,31,32,33,34], our results being in accordance with these data. 

Moroni and collaborators showed that low concentrations of progesterone (from 0.01 up to 1 µM) stimulate A375 melanoma cells proliferation, whereas higher concentrations (10–1000 µM) induce cell density reduction as a result of both cell cycle arrest and apoptosis [40]. Progesterone elicited a dose-dependent inhibitory effect on human melanoma (BLM) cell growth in vitro by inducing autophagy, but estrogen had no inhibitory effect [49]. A similar inhibitory activity of progesterone was observed in mouse melanoma cells—B16F10 [49]. The role of estrogens in melanoma susceptibility and malignancy remained controversial, due to the reported contradictory experimental and clinical findings: estradiol enhances tumor growth and metastasis in B16 melanoma cells, but in human malignant melanoma biopsies, the expressions of estrogen receptors ERα and ERβ are decreased [14]. Several studies described a suppressive role of 17β-estradiol on human SK-Mel-23 melanoma cell (these cells express only ERβ) proliferation [61]. An anti-invasive effect of 17β-estradiol was described in human melanoma cells devoid of ERα receptor [9]. A metabolite of estradiol, 2-methoxyestradiol proved in vitro and in vivo antimelanoma activity [9]. The morphology of A375 and B164A5 melanoma cells following hormone stimulation (1 µM) suffered several changes featured by the round shape of the floating cells that entered apoptosis, whereas the unaffected cells were strongly adherent and similar in shape to the control cells (Figure 10 and Figure 11).

If the cytotoxic profile of test hormones (EE, LNG, and EE + LNG) was similar in human (A375) and murine (B164A5) melanoma cells, the intervention of UVB irradiation determined a different outcome, as follows: (i) in the case of A375 cells, the viability kept the same pattern as after hormones stimulation—a significantly reduced percentage of viable cells (dose-dependent) (Figure 3) and an increased percentage of proapoptotic cells (Figure 4) as compared to UVB-irradiated cells, the strongest effect being recorded for EE + LNG + UVB cells; and (ii) B164A5 cells viability was affected by UVB radiation, but the association of UVB and test hormones led to a lesser cytotoxic effect (Figure 3) and a lower percentage of apoptotic cells (Figure 5) as compared to hormone-only cell stimulation, as UVB made these cells more resistant to hormone cytotoxicity. 

The differences regarding the behavior of A375 and B164A5 melanoma cells after UVB radiation could be attributed to the biological features of each cell line, in terms of: (i) origin: A375—human melanoma cells and B164A5—murine melanoma cells; (ii) morphology: A375 cells present an epithelial morphology and a reduced capacity to determine metastasis, whereas B164A5 cells have a fibroblastic-like morphology and are highly invasive/metastatic, and (iii) melanin content: A375 cells are devoid of melanin while B164A5 cells are melanin-producing cells [62].

Several studies reported a decrease of B16 melanoma cells viability after UVB radiation dependent on the UVB dose [63,64], data that are consistent with our results. Another possible explanation for B164A5 melanoma cells behavior in response to UVB irradiation and hormonal stimulation could be related to melanin, the pigment that is abundantly produced by B164A5 cells. A recent study highlighted the differences at the transcriptomic level between keratinocytes and melanocytes (main UV radiation targets in the skin), melanin representing a key player in the resistance/protection of melanocytes against UVB-induced damage. Melanin is able to counteract the acute effects of UVB radiation on melanocytes by absorbing the radiation. Moreover, it was stated that UV irradiation determined the chemiexcitation of melanin characterized by a continuous release of excited electrons, which has as consequence, DNA-damaged melanocytes long after UV exposure. These data underline an increased susceptibility of keratinocytes to UVB radiation in terms of toxicity as compared to melanocytes [59].

Another mechanism for melanocytes protection against UVB damage or carcinogenesis consists of the development of melanocytic dendrites that act as transporters of melanin pigment from melanocytes to neighboring keratinocytes in response to UVB radiation and hormonal treatment. A similar process of growing dendrites was described in melanoma cells after UV irradiation. Exposure of B16 melanoma cells to a dose of 100 mJ/cm^2^ UVB led to morphological modifications of the cells, characterized by apparition of globular cell bodies and a high number of tree branch-like dendrites [64]. Based on these considerations, we could assume that UVB exposure, together with hormones stimulation of B164A5 melanoma cells led to an increased production of melanin, and to the apparition of dendrites reversing; therefore the cytotoxic effects exerted by the tested hormones in the absence of UVB irradiation, but this hypothesis needs to be further verified. This kind of effect was not observed in A375 melanoma cells due to the lack of melanin in these cells composition.

UVB irradiation ± hormonal treatment induced modifications of melanoma cells morphology (Figure 10 and Figure 11): A375 cells: reduced confluence, round, detached, and floating apoptotic cells; B164A5 cells: round cells, cell shrinkage, and the presence of dendrites. Our data agree with the ones described in the literature that demonstrated that UVB irradiation induced the reorganization of cytoskeletal F-actin with globular cell bodies and a high number of dendrites in B16 melanoma cells [64]. In the presence of hormonal treatment, B164A5 cells began to recover their initial shape (Figure 11), an effect that was also observed in human dermal fibroblasts after stimulation with estrogen [47].

The test hormones were also investigated by an in ovo method to assess qualitatively an irritancy potential after topical application on mucosal or skin tissues. The HET-CAM represents an optimal pre-screening alternative method before animal testing, which is also useful as safety assessment for cutaneous applications [65,66,67]. The evaluation was consonant with in vitro cytotoxic results for the samples unexposed to UVB radiation. LNG showed no irritation both at 1 and 10 µM in consistency, as also indicated by the in vitro low influence on the viability of keratinocytes (HaCaT) and fibroblasts (1BR3). EE alone induced the highest irritation only at the higher tested concentration of 10 µM, but still the effect was very weak compared to the positive control. EE at 1 µM can be considered as non-irritant. The combination of EE + LNG induced, as expected, an even weaker effect at 10 µM, and no irritation at 1 µM. The test hormones are frequently used in micro-doses in transdermal systems or vaginal applications, and they are considered to be non-irritant [68,69]. Moreover, although EE and LNG are associated with vascular risk, in currently prescribed micro-doses does not induce endothelium–dependent vasodilatation [70]. Still, the evaluation of EE, LNG, and their combination in this chorioallantoic membrane environment, can be indicative for the effect on vascular modifications. This may explain why EE stimulates wound healing in in vitro keratinocytes and fibroblasts more than LNG, while, when studied in a vascular assay, LNG seem to attenuate EE effects on the capillary plexus.

## 4. Materials and Methods

### 4.1. Reagents and Cell Lines

Ethinylestradiol (EE) and levonorgestrel (LNG) analytical standards were acquired from Sigma Aldrich (Munich, Germany) and utilized as received. The test compounds (EE, LNG, and their combination—EE + LNG, in a molar ratio of 1:5) were dissolved in DMSO and were stored as stock solutions (5 mM) at 4 °C.

The experiment was conducted using four healthy and two tumor cell lines purchased as frozen items. The healthy cell lines, both human and murine, were as follows: HaCaT—immortalized human keratinocytes (ATCC, LGC Standards GmbH, Wesel, Germany), 1BR3—human skin fibroblast (90011801, ECACC General Collection, Salisbury, UK), HEMa—primary human epidermal melanocytes (ATCC, LGC Standards GmbH), and JB6Cl41-5a—newborn mice epidermis (CRL-2010™, ATCC, LGC Standards GmbH). The tumor cell lines, also human and murine, were: A375—human melanoma (CRL-1619™, ATCC, LGC Standards GmbH) and B164A5—murine melanoma (94042254; Sigma-Aldrich Chemie GmbH, Munich, Germany). All cell lines were kept in standard conditions before culture (liquid nitrogen).

The specific reagents for cell culture such as Dulbecco’s modified Eagle’s medium (DMEM), Eagle’s Minimum Essential Medium (EMEM), Dermal Cell Basal Medium, and Adult Melanocyte Growth Kit were purchased from ATCC (LGC Standards GmbH); non-essential amino acids, fetal bovine serum (FBS), antibiotics mixture (penicillin/streptomycin), phosphate-buffered saline (PBS), trypsin/EDTA and Trypan blue were acquired from Sigma-Aldrich (Munich, Germany).

### 4.2. Cell Culture

Keratinocytes (HaCaT), and human (A375) and murine melanoma (B164A5) cell lines were cultured in DMEM high glucose (4.5 g/L) media, with 15 mM HEPES, and 2 mM l-glutamine supplemented with 10% FCS. A fibroblast (1BR3) cell line was cultured in EMEM supplemented with 15% FBS and for the mice epidermis (JB6 Cl 41-5a) cell line growth was used EMEM supplemented with 0.1% non-essential amino acids and 5% FCS. Primary melanocytes (HEMa) were grown in Dermal Cell Basal Medium supplemented with an Adult Melanocyte Growth Kit. An antibiotic mixture (100 U/mL penicillin, 100 μg/mL streptomycin) was added to all culture media, and the cells were preserved in standard conditions (humidified atmosphere with 5% CO_2_ at 37 °C) and passaged every two days. Cell number was determined using a Countess II FL Automated Cell Counter (AMQAF1000, Thermo Fischer Scientific, Waltham, MA, USA) in the presence of Trypan blue. The cells were seeded in various culture plates (6, 12, and 96 wells) according to the experimental requirements.

### 4.3. UVB Irradiation Protocol

For UVB irradiation experiments, the cells were cultured in 6-/12- and 96-well plates, respectively, and allowed to grow until a confluence of 80–85% was achieved. The protocol consisted of several steps, as follows: the medium was removed prior to UVB exposure to avoid the formation of toxic photoproducts released by the medium [71], and the cells were washed with PBS (phosphate saline buffer); a thin layer of PBS was added in each well. UVB exposure was performed at 312 nm, at a dose of 40 mJ/cm^2^ by means of Biospectra system (Vilber Lourmat, France). Immediately after irradiation, PBS was replaced with culture medium ± test compounds. The stimulation with test compounds (LNG, EE, and EE + LNG) was performed after UVB irradiation.

### 4.4. Cell Viability, Migration and Proliferation Assays

Viability assessment. The viability test applied in the current study was the Alamar blue assay. The cells (1 × 10^4^/200 μL medium/well) were seeded in a 96-well plate and allowed to attach; afterwards, were incubated with different concentrations (1 and 10 μM) of test compounds for 24 h. The absorbance was measured using a xMark™ Microplate Spectrophotometer (BioRad) at 570 nm and 600 nm (reference) wavelengths; and cell viability was calculated according to the method described in our previous studies [72].

Migration and proliferation assay. The migratory character of the cells used in the present study was evaluated by means of a scratch assay, a wound healing type technique. In brief, a number of 2 × 10^5^ cells/well were cultured in 12-well plates, and when the suitable confluence (~90–95%) was reached, a scratch was performed in the middle of the well with a 10 μL sterile tip [73]. To quantify the effect of the test compounds (1 μM EE, LNG, and EE + LNG, respectively) in terms of cell migratory capacity, the difference between the initial and after 24 h wound widths, was determined. Representative images (10× magnification) were recorded by using an Olympus IX73 inverted microscope equipped with DP74 camera (Olympus, Tokyo, Japan) and the wound widths were measured with CellSense Dimension 1.17 (Olympus, Tokyo, Japan). The migration rate was calculated according to the formula described by Felice et al. [74].

Annexin V/PI assay. In order to study the impact of test compounds on cell apoptosis, flow cytometry analysis was performed using an annexin V-FITC apoptosis detection kit (eBioscience, Vienna, Austria). A375 human melanoma and B164A5 murine melanoma cells were seeded into 6-well plates (3 × 10^5^ cells/well) and stimulated with test compounds (1 and 10 µM) for 24 h. After 24 h, the cells were washed with PBS and resuspended in 200 µL Binding Buffer; 5 µL of FITC-conjugated annexin V were added into the cell suspension. Before analysis, 10 µL of propidium iodide solution (20 µg/mL) were added in each sample, followed by 10 min incubation at room temperature in the dark. Cells were analyzed by flow cytometry (FACSCalibur; Becton Dickson, Franklin Lakes, NJ, USA) and unstimulated cells were used as controls. The results were processed using Flowing Software Version 2.5.1 (developed by Perttu Terho, Cell Imaging Core, Turku Centre for Biotechnology, Turun Yliopisto, Finland).

### 4.5. Hen’s Egg Test—Chorioallantoic Membrane (HET-CAM) Assay

The evaluation of hormones biocompatibility and toxicity was assessed in ovo by the Hen’s Egg Chorioallantoic Membrane Test (HET-CAM). The method is applied to evaluate a potential irritant effect of the test compounds on the vascular plexus of the chorioallantoic membrane [65,75]. The HET-CAM method was carried out following ICCVAM recommendations and adapted to our conditions [76]. Thus, the eggs were incubated at 37 °C and controlled humidity. On the third day of incubation (embryonic day of development, EDD 3), 3–4 mL of albumen were extracted in order to facilitate the observation of the chorioallantoic membrane: a hole was cut in the lower part of the egg, which was then covered, and the eggs were reintroduced into the incubator. On EDD 4, a window was cut and removed from the top of the eggs. The hole was then covered, and the eggs were kept in the incubator until EDD 9. Five eggs were used for each tested compound. A volume of 500 µL of control or test solution, respectively, was applied and the modifications produced at the CAM level were monitored by means of stereomicroscopy (Discovery 8 Stereomicroscope, Zeiss, Göttingen, Germany); significant images were recorded (Axio CAM 105 color, Zeiss) before the application and after 5 min of contact with each sample. All images were processed using AxioVision SE64. Rel. 4.9.1 Software (Zeiss), Gimp v 2.8 (https://www.gimp.org/) and ImageJ v 1.50e software (U.S. National Institutes of Health, Bethesda, MD, USA).

The negative control was represented by a phosphate buffer solution (PBS), while the positive control by the sodium dodecyl sulfate (SDS) 1% in PBS. The test compounds were diluted in DMSO at concentrations of 1 µM and 10 µM.

The time needed for the test compounds to induce a particular reaction (hemorrhage—H—blood vessel bleeding, vascular lysis—L—disintegration of blood vessels, coagulation—C—intra or extra-vascular protein denaturizing) was recorded in seconds and was established at 5 min (300 s). The analytical method used to assess the irritant potential of test compounds consisted in calculating the irritation score (IS), using the formula described in our previous study [77]. The formula comprises a factor indicating the impact on vascular damage of the observed effect, e.g., coagulation has the highest impact on irritancy, being represented by a multiplication factor of 9. Therefore, the irritation scores may have values between 0 and 21 [75].

To establish the irritation severity, a severity score (SS) was also calculated. After 5 min of observation, the most pronounced reaction was scored (either hemorrhage, lysis, or coagulation) according to the following scheme: 0 = no reaction; 1 = slight reaction; 2 = moderate reaction; 3 = severe reaction. Mean scores were determined.

### 4.6. Statistical Analysis

The statistical program and software applied in the present study were GraphPad Prism 7 (GraphPad Software, La Jolla, CA, USA), CellSense Dimension 1.17 software, Flowing Software Version 2.5.1, AxioVision SE64. Rel. 4.9.1 Software, Gimp v 2.8 and ImageJ v 1.50e software. Data were analyzed using paired Student’s *t* tests or one-way ANOVA, followed by Tukey’s post-tests when appropriate, to determine the statistical difference between experimental and control groups; *, **, *** and **** indicate *p* < 0.05, *p* < 0.01, *p* < 0.001 and *p* < 0.0001, respectively.

## 5. Conclusions

This initial study that evaluates the potential antimelanoma activity of ethinylestradiol, levonorgestrel, and their combination ± UVB irradiation—proposed an approach that might prove its utility in further studies. The two hormones (EE and LNG) and their combination (EE + LNG) did not interfere with human and murine healthy skin cell viability at the lowest concentration tested, whereas in the case of melanoma cells, this concentration induced a significant cytotoxic effect. By increasing the concentration of hormones and adding UVB irradiation, the cytotoxicity induced was at a higher extent in all healthy cell lines and in human melanoma cells—A375. In the case of murine melanoma cells—B164A5, the association of hormones and UVB stress led to an increase of viable cell percentage and a decrease of early apoptotic cells, a possible key role being played by melanin. In ovo experiments confirmed the harmless activity of the hormones at low doses, albeit a higher concentration was responsible for a weak irritant effect (for EE and EE + LNG). These experimental observations offer a reliable background for further in vitro studies to define the mechanisms involved in cell type-dependent antimelanoma activity exerted by the two hormones, and to explain the possible role of melanin in the protection of melanoma cells against hormonal treatment.

## Figures and Tables

**Table 1:** The irritant potential of tested hormones: EE, LNG, EE + LNG.

Test Compound and Controls	Irritation Score (Mean)	Irritation Severity (Mean)	Classification of the Effect
PBS Negative control	0 ± 0	0 ± 0	Non-irritant
SDS Positive control	15.07 ± 1.08	2.67 ± 0.58	Strong irritant
DMSO 1% Solvent Control	0 ± 0	0 ± 0	Non-irritant
EE 1 µM	0 ± 0	0 ± 0	Non-irritant
EE 10 µM	2.79 ± 0.55	1.33 ± 0.58	Weak irritant
LNG 1 µM	0 ± 0	0 ± 0	Non-irritant
LNG 10 µM	0 ± 0	0 ± 0	Non-irritant
EE + LNG 1 µM	0.63 ± 0.3	0.83 ± 0.29	Non-irritant
EE + LNG 10 µM	1.23 ± 0.3	1 ± 0	Weak irritant

## Supplementary Materials

The supplementary materials are available online at http://www.mdpi.com/1422-0067/19/11/3600/s1.

## Abbreviations

A375	Human melanoma cells
B164A5	Murine melanoma cells
BCC	Basal cell carcinoma
1BR3	Human skin fibroblasts
DMSO	Dimethyl sulfoxide
EE	Ethinylestradiol
ER	Estrogen receptor
ET-1	Endothelin-1
HaCaT	Human immortalized keratinocytes
HEMa	Human primary epidermal melanocytes
HET-CAM	Hen’s Egg Test—chick chorioallantoic membrane assay
ICCVAM	Interagency Coordinating Committee on the Validation of Alternative Methods
IS	Irritation score
JB6Cl415a	Newborn mice epidermis
LNG	Levonorgestrel
MSH	Melanocyte-stimulating hormone
PBS	Phosphate saline buffer
SCC	Squamous cell carcinoma
SCF	Stem cell factor
SDS	Sodium dodecyl sulfate
SS	Severity score
UVB	Ultraviolet B radiation
UVA	Ultraviolet A radiation
