# Supplementary material for: Ethinylestradiol and Levonorgestrel as Active Agents in Normal Skin, and Pathological Conditions Induced by UVB Exposure: In Vitro and In Ovo Assessments
Source: Int J Mol Sci. 2018 Nov 14;19(11):3600. doi: 10.3390/ijms19113600 (PMC6275072; doi:10.3390/ijms19113600)
Supplement: Supplementary file 1 [file ijms-19-03600-s001.pdf]

Ethinylestradiol and Levonorgestrel, as Active Agents in Normal Skin and Pathological Conditions Induced by UVB Exposure: In Vitro and In Ovo Assessment

Dorina Coricovac [1,†], Claudia Farcas [1,†], Cristian Nica [2], Iulia Pinzaru [1,*], Sebastian Simu [1], Dana Stoian [2], Codruta Soica [1], Maria Proks [1], Stefana Avram [1], Dan Navolan [2], Catalin Dumitru [2], Ramona Amina Popovici [3], Cristina Adriana Dehelean [1]

Supplementary Material

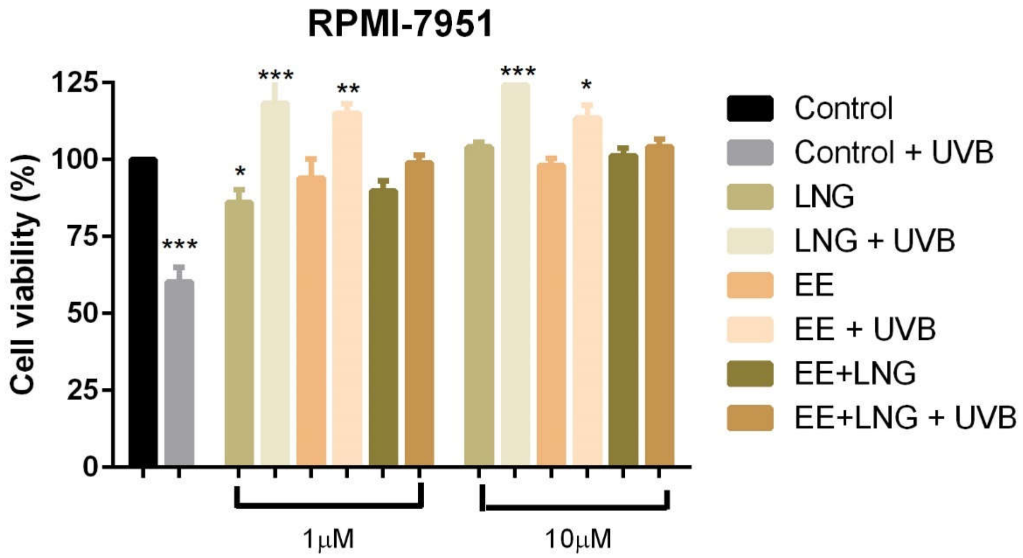

Figure S1. The effect of test compounds (1 and 10 μM) ± UVB irradiation on RPMI-7951 cells viability at 24 hr post-stimulation. The results are expressed as cell viability percentage (%) normalized to control cells. The data represent the mean values ± SD of three independent experiments. One-way ANOVA analysis was applied to determine the statistical differences followed by Tukey's multiple comparisons test (* p <0.05; ** p<0.01; *** p<0.001, **** p<0.0001).

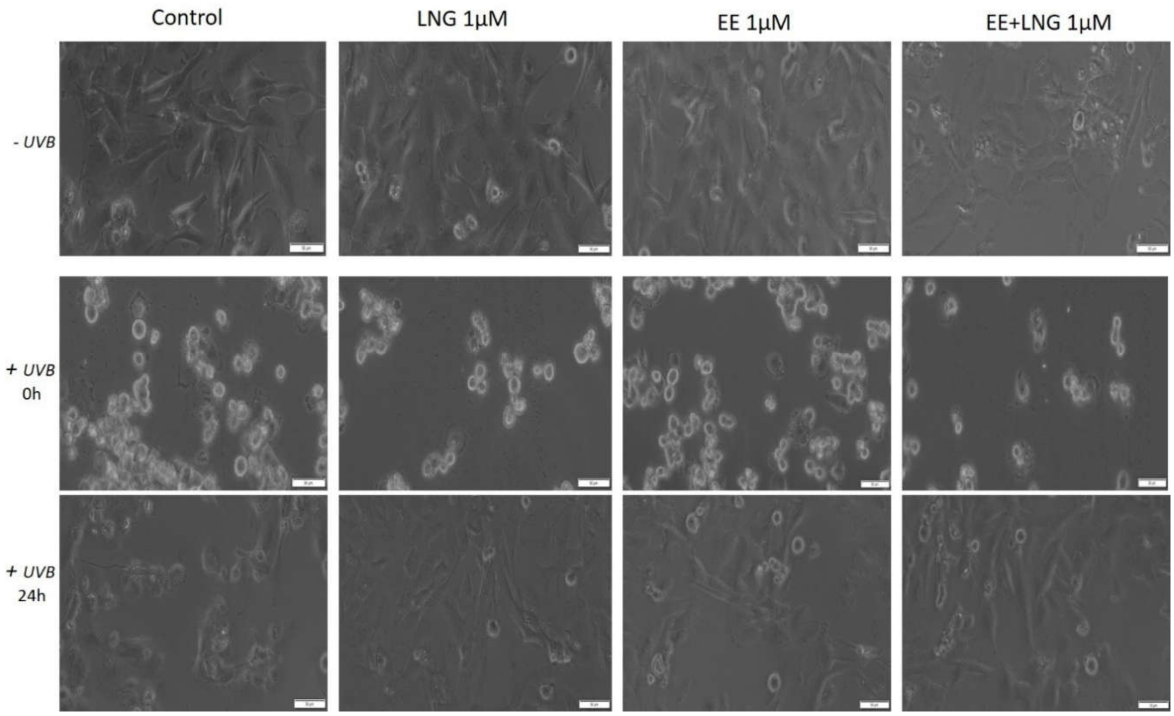

Figure S2. In vitro morphological aspect of human melanoma cells—RPMI-7951 cells, stimulated with levonorgestrel (LNG), ethinylestradiol (EE), and an ethinylestradiol/levonorgestrel combination (EE+LNG), respectively, at a concentration of 1 µM ± UVB irradiation. Scale bars represent 50 µM.

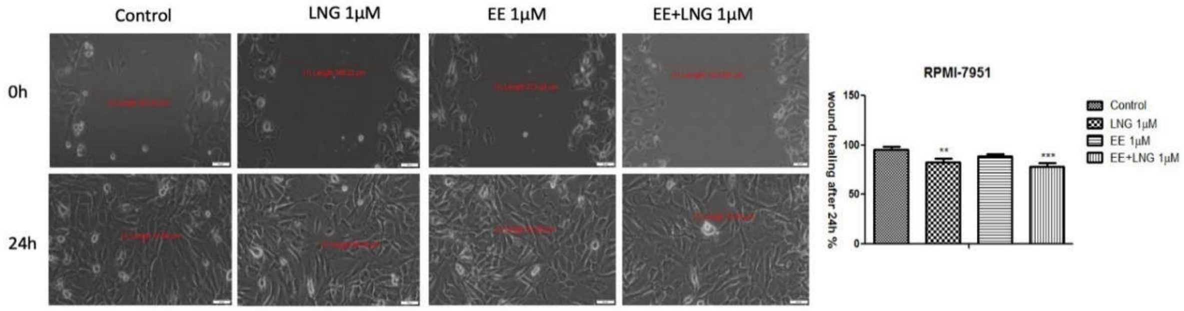

Figure S3. The impact of test compounds (LNG, EE, and EE+LNG – 1 µM) on the migratory capacity of human melanoma cells – RPMI-7951. Wound closure was recorded by bright field microscopy initially—0 hr and after 24 hr, respectively. Scale bars represent 50 µm. The bar graphs are expressed as percentage of wound closure after 24 hr compared to the initial surface. The data represent the mean values ± SD of three independent experiments. One-way ANOVA analysis was applied to determine the statistical differences followed by Tukey post-test (* $p < 0.05$; ** $p<0.01$; *** $p<0.001$ vs control—no stimulation).
